# Association between brain volume and disability over time in multiple sclerosis

**DOI:** 10.1177/20552173221144230

**Published:** 2022-12-18

**Authors:** Thomas Moridi, Leszek Stawiarz, Kyla A McKay, Benjamin V Ineichen, Russell Ouellette, Daniel Ferreira, J-Sebastian Muehlboeck, Eric Westman, Ingrid Kockum, Tomas Olsson, Fredrik Piehl, Jan Hillert, Ali Manouchehrinia, Tobias Granberg

**Affiliations:** Department of Clinical Neuroscience, 27106 Karolinska Institutet, Stockholm, Sweden; Center for Molecular Medicine, 59562 Karolinska University Hospital, Stockholm, Sweden; Department of Clinical Neuroscience, 27106 Karolinska Institutet, Stockholm, Sweden; Department of Clinical Neuroscience, 27106 Karolinska Institutet, Stockholm, Sweden; Division of Neuroradiology, Department of Radiology, 59562 Karolinska University Hospital, Stockholm, Sweden; Division of Clinical Geriatrics, Center for Alzheimer Research, Department of Neurobiology, Care Sciences and Society, 27106 Karolinska Institutet, Stockholm, Sweden; Department of Clinical Neuroscience, 27106 Karolinska Institutet, Stockholm, Sweden; Center for Molecular Medicine, 59562 Karolinska University Hospital, Stockholm, Sweden; Department of Clinical Neuroscience, 27106 Karolinska Institutet, Stockholm, Sweden; Center for Molecular Medicine, 59562 Karolinska University Hospital, Stockholm, Sweden; Center for Neurology, Academic Specialist Center, Stockholm Healthcare Services, Stockholm, Sweden; Department of Clinical Neuroscience, 27106 Karolinska Institutet, Stockholm, Sweden; Department of Neurology, 59562 Karolinska University Hospital, Stockholm, Sweden; Department of Clinical Neuroscience, 27106 Karolinska Institutet, Stockholm, Sweden; Center for Molecular Medicine, 59562 Karolinska University Hospital, Stockholm, Sweden; Department of Clinical Neuroscience, 27106 Karolinska Institutet, Stockholm, Sweden; Division of Neuroradiology, Department of Radiology, 59562 Karolinska University Hospital, Stockholm, Sweden

**Keywords:** brain atrophy, disability, magnetic resonance imaging, multiple sclerosis

## Abstract

**Background:**

Most previous multiple sclerosis (MS) brain atrophy studies using MS impact scale 29 (MSIS-29) or symbol digit modalities test (SDMT) have been cross-sectional with limited sets of clinical outcomes.

**Objectives:**

To investigate which brain and lesion volume metrics show the strongest long-term associations with the expanded disability status scale (EDSS), SDMT, and MSIS-29, and whether MRI-clinical associations vary with age.

**Methods:**

We acquired MRI and clinical data from a real-world Swedish MS cohort. FreeSurfer and SPM Lesion Segmentation Tool were used to obtain brain parenchymal, cortical and subcortical grey matter, thalamic and white matter fractions as well as T_1_- and T_2_-lesion volumes. Mixed-effects and rolling regression models were used in the statistical analyses.

**Results:**

We included 989 persons with MS followed for a median of 9.3 (EDSS), 10.1 (SDMT), and 9.3 (MSIS-29) years, respectively. In a cross-sectional analysis, the strength of the associations of the MRI metrics with the EDSS and MSIS-29 was found to drastically increase after 40–50 years of age. Low baseline regional grey matter fractions were associated with longitudinal increase of EDSS and physical MSIS-29 scores and decrease in SDMT scores and these atrophy measures were stronger predictors than the lesion volumes.

**Conclusions:**

The strength of MRI-clinical associations increase with age. Grey matter volume fractions are stronger predictors of long-term disability measures than lesion volumes.

## Introduction

Multiple sclerosis (MS) is a heterogeneous neuroinflammatory and neurodegenerative disease of the central nervous system (CNS), which represents one of the main causes of neurologic disability among young adults.^1^ Magnetic resonance imaging (MRI) lesion load is the only widely accepted paraclinical biomarker for inflammatory disease activity and treatment response in MS in the clinical setting.^2^ T_2_-lesion load has also been shown to be moderately predictive of future disability progression.^3,4^ More recent studies, however, suggest that brain atrophy more closely reflects neurodegeneration and subsequent cognitive and physical disability progression than lesion load in MS.^5–8^

Persons with MS display more brain volume loss over time than healthy controls.^9^ The brain volume loss also differs within the MS population, being more pronounced for the progressive disease phenotypes, and individuals with a high predicted brain age exhibit a higher level of clinical progression.^8,10^ In particular, grey matter volumes are reduced compared with other brain regions and are also more strongly associated with physical disability measured with the expanded disability status scale (EDSS), than other regional brain atrophy measures or the T_2_ lesion volume.^6,11^ Importantly, brain atrophy is also associated with deficits in cognitive functions, including information processing speed as measured with the symbol digit modalities test (SDMT).^7,12,13^ Cortical and subcortical grey matter, in particular thalamic atrophy, have been suggested to be the best correlates of cognitive function compared with other atrophy and lesion metrics, although only a few studies have specifically focused on information processing speed.^7,14,15^ In addition to these objective measures of physical and cognitive disability, self-reported burden of disease may provide pivotal information when gauging the impact of MS at the individual level. The MS impact scale 29 (MSIS-29) on physical and psychological well-being correlates with clinical measures of disease severity, but its relationship with brain atrophy is largely unknown.^16,17^

While large high-quality longitudinal studies of MRI-EDSS associations have been performed previously, most observational studies of the SDMT and MSIS-29 have been cross-sectional and/or conducted in small cohorts with limited sets of MRI and clinical measures.^6,8^ Furthermore, while the correlations between clinical severity and brain or lesion volumes or estimated brain age have been studied before, the potential variation of the strength of these MRI-clinical associations across different age groups has, to the best of our knowledge, not been assessed before. Therefore, we aimed to investigate the following in a large real-world Swedish MS cohort:

- Whether or not the strength of associations between MRI measures (including brain parenchymal fraction, cortical grey matter fraction, subcortical grey matter fraction, thalamic fraction, white matter fraction, T_1_- and T_2_-lesion volumes) and clinical variables (including EDSS, SDMT, and physical/psychological MSIS-29 scores) vary with age in a cross-sectional analysis at baseline and at which ages these potential variations occur.
- Whether or not these baseline MRI metrics are associated with longitudinal EDSS, SDMT, and physical/psychological MSIS-29 scores, and which of these MRI metrics show the strongest associations with the clinical scores.

## Methods

### Study population

The data used in this study was obtained from the Epidemiological Investigation of MS (EIMS), Genes and Environment in MS (GEMS), Immunomodulation and MS Epidemiology (IMSE), and Stockholm Prospective Assessment of MS (STOP-MS) prospective cohort studies in which persons with MS had been recruited consecutively between 2001 and 2015 upon their visits at the neurology clinics in Stockholm County, Sweden. MS was diagnosed according to the 2017 McDonald criteria.^18^ These criteria were also used retrospectively for persons diagnosed with MS prior to 2017 to ensure consistent inclusion criteria across for the entire cohort. Study participants were followed with clinical scores and MRI on an approximately annual basis. The sampling of MRI scans with the scanning protocol used in this study ended in 2015, while sampling of clinical scores is still ongoing. Extraction of the clinical score data for the current study was performed in 2020. The EIMS, GEMS, IMSE, and STOP-MS cohorts have been described in more detail previously.^19–22^ The inclusion criteria of the current study were that persons must have had at least one MRI scan with volumetric output and one EDSS, SDMT, or MSIS-29 score following an MS diagnosis, and the first MRI must not have been performed more than 6 months after sampling of the first clinical score. Persons with missing clinical data, including age, age at disease onset, sex, or MS subtype were excluded. Persons who were lost to follow-up were not excluded from the study. No further inclusion or exclusion criteria were applied. Ethical permits for these studies have been obtained from the Regional Ethics Review Board of Stockholm and all study participants have given written informed consent to participate.

### Clinical measures

The EDSS is a validated measure of neurological disability ranging from 0 (no disability) to 10 (death due to MS).^23^ Information processing speed was measured using the SDMT which has been validated for use in the MS population.^24^ SDMT scores range from 0 to 110, with a higher score indicating better performance in information processing speed. The MSIS-29 is a self-reported measure of the burden of disease on physical and psychological health.^16^ The resulting scores range from 0 to 5, with a high score indicating a more detrimental impact of the disease. Time of conversion to secondary progressive MS was determined by the treating physician's assessment of the medical history and by neurological examinations. All clinical data were obtained from the Swedish MS registry, a nationwide database comprising clinical information from 64 neurology clinics in Sweden.

### MRI acquisition and processing

3D T_1_-weighted MRI scans were performed on three 1.5 Tesla scanners and one 3 Tesla scanner (Aera, Avanto, Vision Plus and Trio; Siemens Healthcare, Erlangen, Germany) using a magnetization-prepared rapid gradient echo sequence (MPRAGE) with 1.5 × 1.0 × 1.0 mm spatial resolution. The scanning protocol was consistent across all scans. FreeSurfer 6.0.0 (https://surfer.nmr.mgh.harvard.edu/) was used for automatic segmentation of the T_1_-lesion volume and the volumes of the brain parenchyma, cortical and subcortical grey matter, thalamus and white matter, normalized to the estimated total intracranial volume resulting in corresponding brain fractions.^25^ No lesion-filling technique was used prior to the brain segmentation. The brain segmentation was performed using a subject template in a common space and then each time point was registered to this template to perform an unbiased analysis.^25^ The Lesion Segmentation Tool (https://www.statistical-modelling.de/lst.html) with the Lesion Prediction Algorithm for SPM was used for automated segmentation of the T_2_ lesion volume using either 2D or 3D fluid-attenuated inversion recovery (FLAIR) sequences.^26^ The results were visually inspected by a trained neuroradiologist. Further technical information about the image acquisition is presented in Supplementary Table 1.

### Statistical analysis

All statistical analyses were performed in R version 4.0.3. In order to obtain comparable effect sizes across all models, both the MRI predictor variables and the clinical outcome variables were *z*-scored. Since the T_1_- and T_2_-lesion volume data were heavily skewed, rank-based inverse normal transformation was used to attain a normal distribution.

In order to assess how the MRI measures associated with the clinical variables across different ages of the study participants at baseline, rolling multiple regression models were used with the tibbletime package in R.^27^ The rolling regression performs linear regression on a moving window of data points, for instance, a window of 30 persons moving from the lowest to the highest ages of the persons while adjusting for covariates. We also assessed the associations between the baseline MRI and longitudinal clinical variables with linear mixed-effects models. The outcome was defined as the change in the clinical score over time for each unit of change in the baseline MRI predictor variable while adjusting for covariates. The predictor was categorized as *high* or *low* depending on whether the value was above or below/equal to the median of the study population.

The false discovery rate (FDR) method was used to adjust *p*-values for multiple testing using *p* < 0.05 as a cut-off for statistical significance. Further details about the statistical analyses, covariate selection, and data availability are described in Appendix I.

## Results

### Clinical characteristics

**Figure 1.**
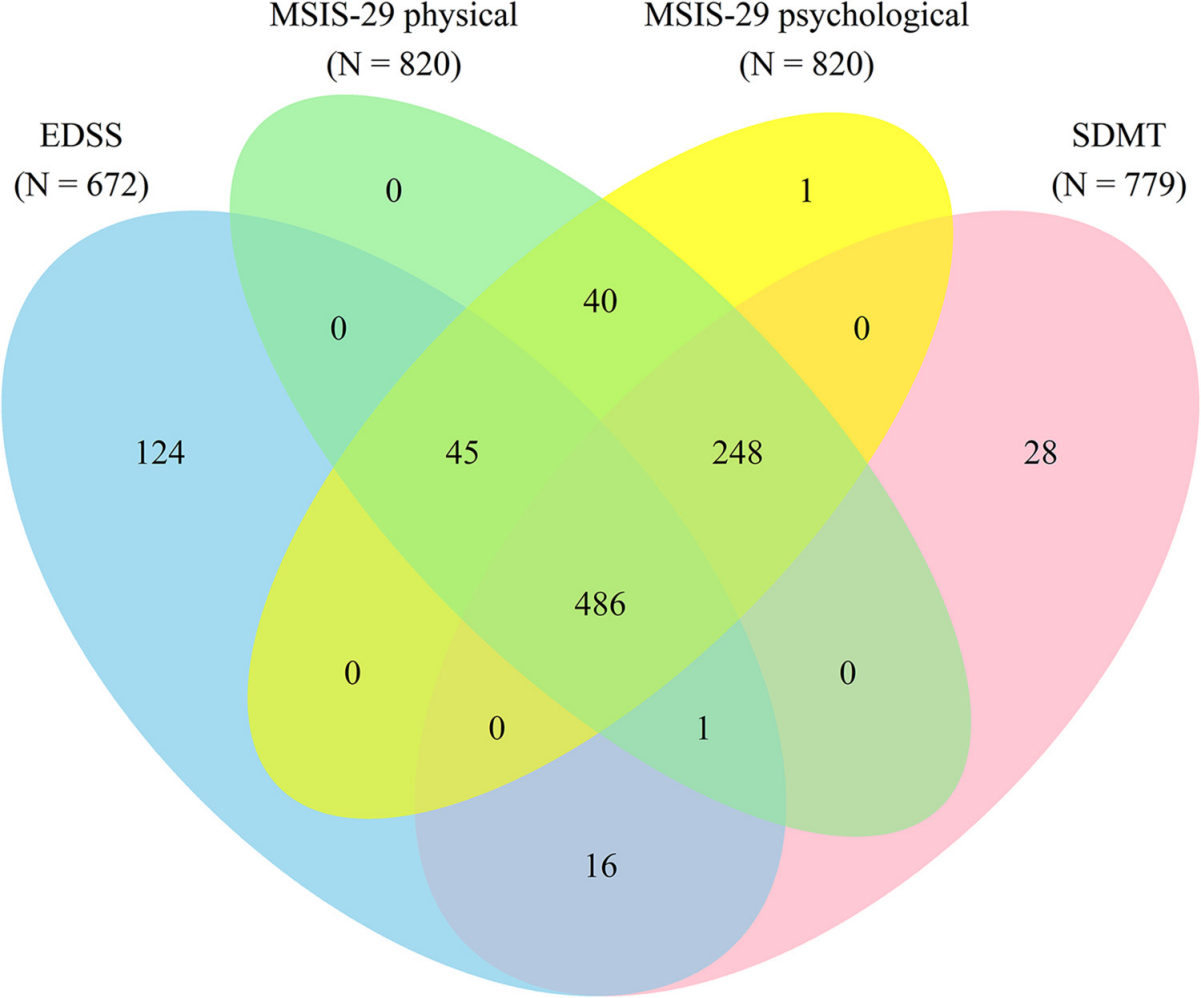
Venn diagram of the overlap of available clinical data in the study cohort. Abbreviations: EDSS, expanded disability status scale; MSIS-29, multiple sclerosis impact scale 29; SDMT, symbol digit modalities test.

The clinical characteristics of the study population are presented in Table 1. The overlap of available clinical data in the study cohort is shown in Figure 1 and a flowchart of inclusion/exclusions and missing data is shown in Supplementary Figure 1. Out of a total of 989 persons with MS with at least one MRI scan (3461 scans in total), there were 672 persons with a minimum of one recorded EDSS score; 779 persons with an SDMT score; and 820 persons with an MSIS-29 physical and/or psychological score that were included in the final analysis. The clinical characteristics for each of these subcohorts were very similar (Table 1), indicating that the disparity in availability of EDSS, SDMT, and MSIS-29 scores did not confer any substantial degree of bias to the results of this study.

**Table 1.** Clinical characteristics of the multiple sclerosis study population.

**Patient variables**	**EDSS (*N* = 672)**	**SDMT (*N* = 779)**	**MSIS-29 physical (*N* = 820)**	**MSIS-29 psychological (*N* = 820)**
Clinical variable at baseline, median (IQR)	2.0 (1.0–3.0)	47 (36–54)	1.70 (1.25–2.70)	2.22 (1.56–3.03)
Sex at baseline, % females/males	70/30	71/29	71/29	71/29
Age at baseline MRI scan, median (IQR)	37.1 (28.8–46.8)	35.3 (28.3–43.3)	35.9 (28.5–44.1)	35.9 (28.5–44.0)
Age at baseline clinical examination, median (IQR)	38.2 (29.9–48.3)	40.8 (32.4–49.9)	39.6 (30.9–48.8)	39.6 (30.9–48.8)
Age at onset, median (IQR)	32.9 (26.0–41.2)	30.2 (24.5–37.4)	30.6 (24.8–38.1)	30.6 (24.8–38.1)
Diagnosis at baseline, No. RR/SP/PR/PP	578/42/12/40	687/58/14/20	716/57/16/31	715/58/16/31
Age at conversion to secondary progressive multiple sclerosis, median (IQR)*	50 (43–58)	48 (39–59)	47 (38–56)	47 (39–56)
Years between adjacent clinical scores, median (IQR)	0.92 (0.50–1.4)	0.52 (0.46–0.86)	0.64 (0.48–1.0)	0.64 (0.48–1.0)
Patients with any platform DMT prior to first clinical score (%)	32	78	75	75
Patients with any highly active DMT prior to first clinical score (%)	4	19	15	15
Patients with any platform DMT prior to last clinical score (%)	76	84	83	83
Patients with any highly active DMT prior to last clinical score (%)	54	71	67	67
No. of scans at baseline for Aera/Avanto/Trio/Vision Plus	67/247/124/234	96/295/137/251	95/281/135/309	95/281/135/309
2D/3D FLAIR, No. at baseline	557/215	628/151	666/154	666/154
Whole brain fraction at baseline, median (IQR)	0.74 (0.73–0.76)	0.74 (0.73–0.76)	0.74 (0.73–0.76)	0.74 (0.73–0.76)
Cortical grey matter fraction at baseline, median (IQR)	0.30 (0.29–0.32)	0.30 (0.29–0.32)	0.30 (0.29–0.32)	0.30 (0.29–0.32)
Subcortical grey matter fraction at baseline, median (IQR)	0.036 (0.034 –0.038)	0.036 (0.034 –0.038)	0.036 (0.034–0.038)	0.036 (0.034–0.038)
Thalamic fraction at baseline, median (IQR)	0.0093 (0.0087–0.010)	0.0092 (0.0086–0.0099)	0.0092 (0.0086–0.0099)	0.0092 (0.0086–0.0099)
White matter fraction at baseline, median (IQR)	0.30 (0.28–0.31)	0.30 (0.28–0.31)	0.30 (0.28–0.31)	0.30 (0.28–0.31)
T_1_-lesion volume at baseline, median (IQR), mL	1.8 (1.1–3.4)	1.8 (1.1–3.5)	1.9 (1.2–3.5)	1.9 (1.2–3.5)
T_2_-lesion volume at baseline, median (IQR), mL	4.0 (1.7–16)	3.8 (1.5–14)	3.8 (1.5–13)	3.8 (1.5–13)

Abbreviations: DMT, disease modifying treatment; EDSS, expanded disability status scale; FLAIR, fluid-attenuated inversion recovery; IQR, interquartile range; MSIS-29, multiple sclerosis impact scale 29; PP, primary progressive; PR, progressive-relapsing; RR, relapsing-remitting; SDMT, symbol digit modalities test; SP, secondary progressive.

*This refers to the subcohorts that only included individuals with ≤ 6 months between baseline MRI and clinical score.

For the association analysis of the baseline MRI measures with the longitudinal clinical outcomes, persons were followed with the EDSS for a median of 9.3 (IQR 6.2–13.7) years after the first MRI and completed a median of 7 (IQR 4–10) scores. For the SDMT, persons were followed for a median of 10.1 (IQR 6.5–14.0) years and completed a median of 6 (IQR 3–11) scores. For the MSIS-29 physical and psychological, persons were followed for a median of 9.3 (IQR 5.4–13.8) years and completed a median of 5 (IQR 3–10) scores.

### Associations between baseline MRI measures and clinical variables across age

**Figure 2.**
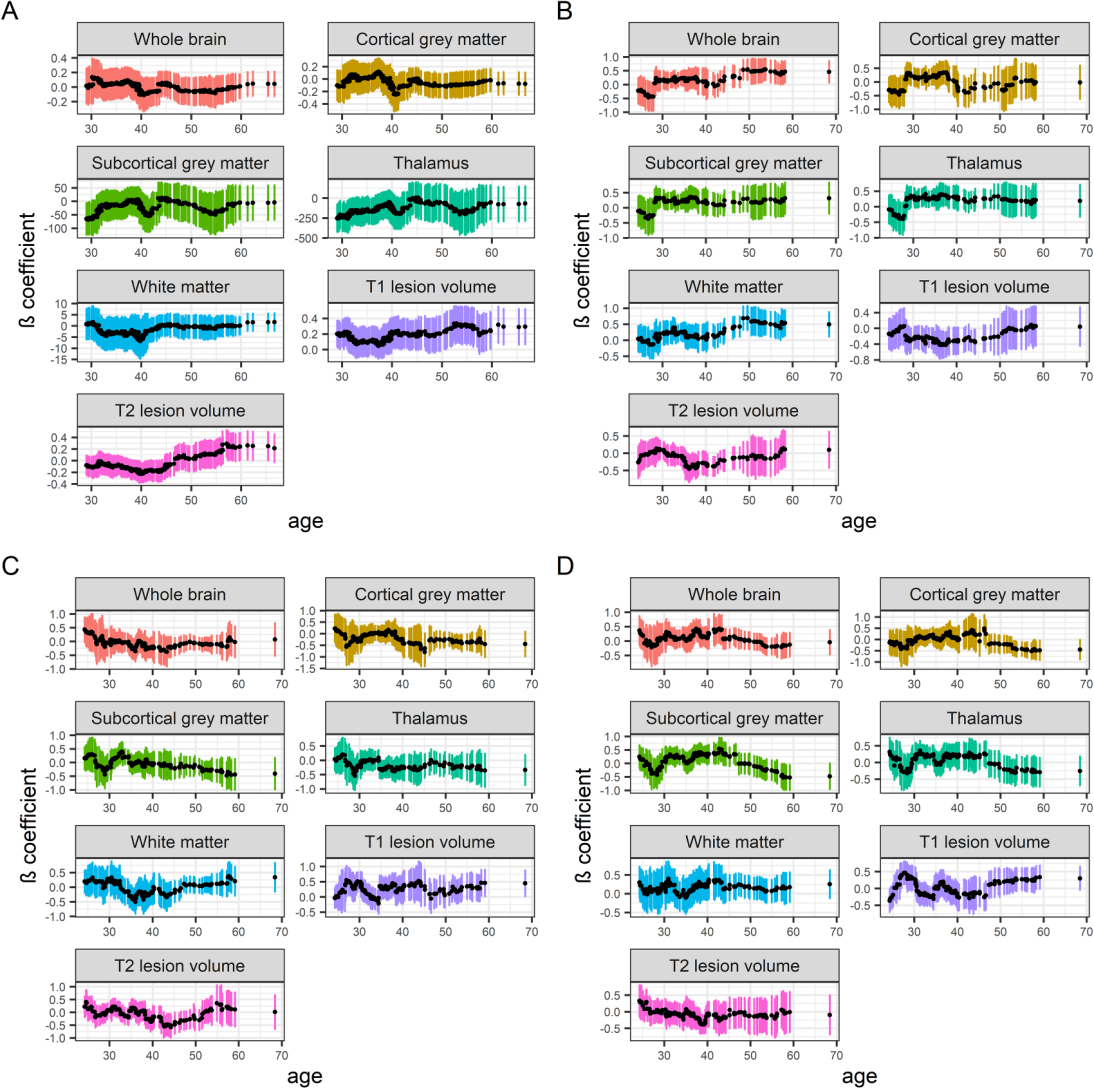
Rolling regression models of baseline MRI measures and clinical variables across ages. Rolling multiple regression models of how baseline MRI brain volume fractions and lesion volumes associate with clinical variables across different ages of the persons with MS. Due to a larger sample size, a bandwidth of 100 persons was used for the EDSS rolling regressions, while a bandwidth of 30 persons was used for the other clinical variables. Stepwise reduction of multiple regression models including the entire age span of persons was used for covariate selection. MS subtype (relapsing-onset vs primary progressive MS) was not included as a covariate due to too few cases of primary progressive MS. The colored vertical bars represent the 95% confidence intervals for each regression. Abbreviations: DMT, Disease-modifying treatment; EDSS, expanded disability status scale; MRI, magnetic resonance imaging; MS, multiple sclerosis; MSIS-29, MS impact scale 29; SDMT, symbol digit modalities test. (a) EDSS. *N* = 441. Age at onset was used as a covariate. (b) SDMT. *N* = 148. MRI scanner was used as a covariate. (c) MSIS-29 physical. *N* = 190. Age at onset, sex, MRI scanner and platform DMT exposure were used as covariates. (d) MSIS-29 psychological. *N* = 190. MRI scanner was used as a covariate.

We assessed how cross-sectional baseline MRI/clinical associations varied across the age span of the study participants. We included individuals with no more than 6 months between the first scan and clinical examination. The predictors used in these models are presented in Appendix II. Rolling regression models showed that the EDSS was more strongly associated with the MRI measures at old ages, with inflection points at 40–50 years of age, except for the white matter fractions, which had the strongest negative associations at 30–40 years of age (Figure 2). MSIS-29 physical, and especially psychological, scores showed similar association patterns across ages as the EDSS. The inflection points for a stronger association with the EDSS and MSIS-29 coincided with the ages that the study participants clinically converted to secondary progressive MS (Table 1). For the atrophy measures, the direction and strength of the SDMT associations differed between measures and across ages with no obvious pattern. For the T_1_- and T_2_-lesion volumes, the SDMT associations were the strongest between 30 and 50 years of age. As a sensitivity analysis, we excluded persons with primary progressive MS and persons with a recorded relapse within 6 months before the clinical scores in the rolling regression analysis. In this sensitivity analysis, the differences in the effect sizes between different ages were overall smaller compared with the main analysis, except the EDSS that remained substantially more strongly associated with the T_2_-lesion volume at old ages than at young ages (Supplementary Figure 2).

### Associations between baseline MRI measures and longitudinal clinical variables

**Figure 3.**
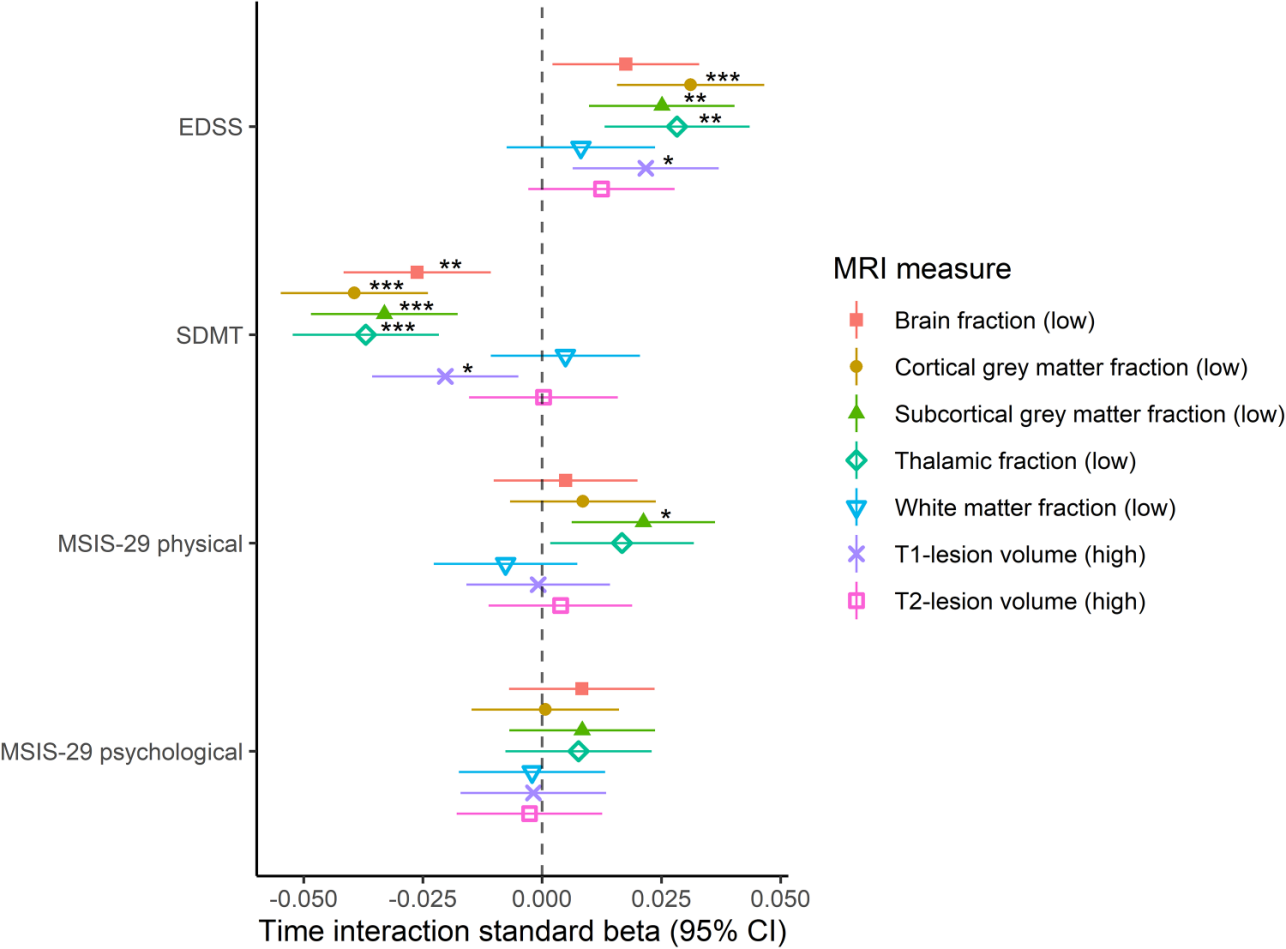
Forest plot of linear mixed-effects models of longitudinal clinical measures and baseline MRI measures with time interaction. In all models, study subjects and MRI scanners were used as nested random effects with random slopes at time of measurement of the clinical scores. The asterisks represent *p*-values after false discovery rate correction. *p < 0.05; **p < 0.01; ***p < 0.001. No asterisk(s)*p* ≥ 0.05. The CIs were not corrected for multiple comparisons. Abbreviations: CI, confidence interval; DMT, disease-modifying treatment; EDSS, expanded disability status scale; FLAIR, fluid-attenuated inversion recovery; MRI, magnetic resonance imaging; MSIS-29, multiple sclerosis impact scale 29; SDMT, symbol digit modalities test. *EDSS*: *N* = 672. There were 5515 EDSS scores. Baseline age at MRI, age at onset, disease type (relapsing-onset vs primary progressive multiple sclerosis), highly active DMT exposure, MRI measure, time after baseline clinical examination and an MRI measure*time interaction term were used as fixed effects. *SDMT*: *N* = 779. There were 6011 SDMT scores. Baseline age at MRI, age at onset, total number of completed SDMTs, FLAIR sequence type (2D vs 3D), disease type (relapsing-onset vs primary progressive multiple sclerosis), highly active DMT exposure, MRI measure, time after baseline clinical examination and an MRI measure*time interaction term were used as fixed effects. *MSIS-29 physical*: *N* = 820. There were 5497 MSIS-29 physical scores. Baseline age at MRI, baseline age at clinical examination, age at onset, sex, FLAIR sequence type (2D vs 3D), disease type (relapsing-onset vs primary progressive multiple sclerosis), platform and highly active DMT exposure, MRI measure, time after baseline clinical examination and an MRI measure*time interaction term were used as fixed effects. *MSIS-29 psychological*: *N* = 820. There were 5497 MSIS-29 psychological scores. Baseline age at MRI, age at onset, sex, disease type (relapsing-onset vs primary progressive multiple sclerosis), platform and highly active DMT exposure, MRI measure, time after baseline clinical examination, and an MRI measure*time interaction term were used as fixed effects.

**Figure 4.**
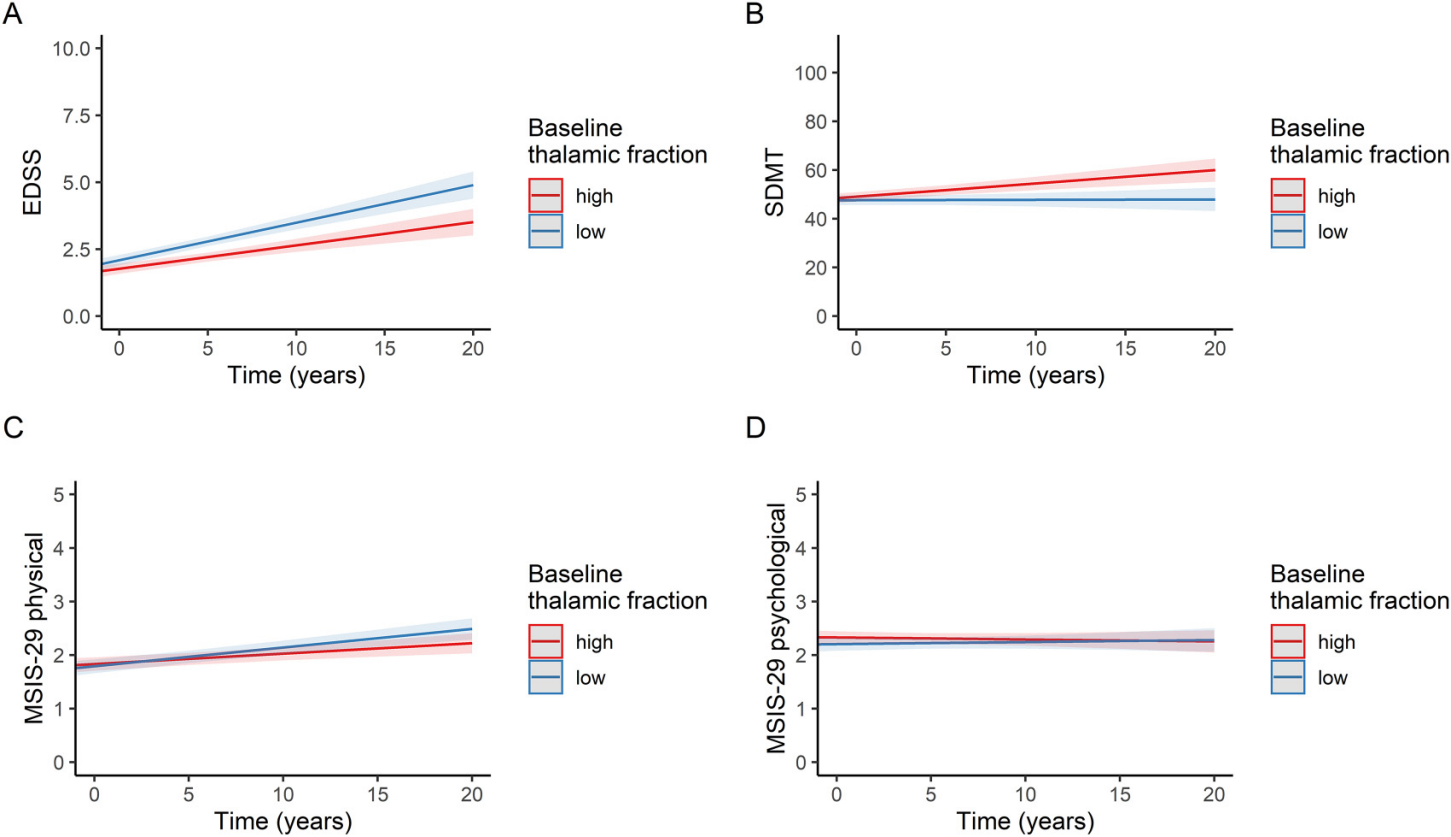
Longitudinal clinical scores were categorized by baseline thalamic fractions. The straight red and blue lines are fitted from linear mixed-effects models and represent individuals with “high” and “low” baseline thalamic fractions, respectively. The shaded red and blue areas represent 95% confidence intervals. (a) EDSS, (b) SDMT, (c) MSIS-29 physical, (d) MSIS-29 psychological. Abbreviations: EDSS, expanded disability status scale; MSIS-29, multiple sclerosis impact scale 29; SDMT, symbol digit modalities test.

To assess how the baseline MRI measures were associated with the future rate of change in clinical outcomes, we used linear mixed-effects models with an interaction term for time (from the baseline MRI scan to each clinical outcome measurement). The predictors used in these models are presented in Appendix II. The time interaction terms showed that there was a significant increase of 0.031, 0.025, and 0.028. EDSS score standard deviations (corresponding to an increase of 0.059, 0.047, and 0.053 in raw EDSS scores) per year in the *low* volume group compared to the *high* volume group for the cortical grey matter, subcortical grey matter, and thalamic volume fractions, respectively (Figure 3). There was an annual decrease of 0.026, 0.039, 0.033, and 0.037 SDMT score standard deviations (corresponding to a decrease of 0.38, 0.56, 0.47, and 0.53 in raw SDMT scores) in the *low* volume group compared to the *high* volume group for the brain parenchymal, cortical grey matter, subcortical grey matter, and thalamic volume fractions, respectively. *High* baseline T_1_-lesion volumes were also associated with deterioration in the EDSS (0.022 standard deviations [0.041 in raw EDSS scores] per year) and SDMT (−0.020 standard deviations [−0.29 in raw SDMT scores] per year) scores over time, although the effect sizes were smaller than the corresponding effect sizes for the cortical grey matter, subcortical grey matter, and thalamic volume fractions. Apart from an association between *low* subcortical grey matter volume fractions and increased MSIS-29 physical scores (0.021 standard deviations/year), none of the baseline MRI measures were significantly associated with the rate of change of MSIS-29 physical or psychological scores. As an illustrative example, Figure 4 displays the trajectories of the clinical scores categorized according to baseline thalamic volume fractions. The results of the main analysis displayed in Figure 3 were similar to sensitivity analyses that (1) only included individuals with all four clinical variables available and (2) only included the MRI scanner Vision, for which the largest number of clinical data points were available (Supplementary Figures 3 and 4, respectively).

## Discussion

In this study, we investigated the relationship of different atrophy and lesion burden MRI-metrics with clinical outcomes and age in a large prospective real-world cohort of persons with MS and long-term follow-up. Previous studies have indicated that brain atrophy is more strongly correlated with physical and cognitive disability than lesion burden.^5–8^ However, the relative strength of the associations for different structures in the brain and how these associations vary with age was less known.

The rolling regression models of the MRI measures and the clinical variables across age suggest that the associations differ in strength at different ages of the persons with MS. To the best of our knowledge, this is the first assessment of how the strength of the associations between MRI metrics and clinical outcomes varies with age. For physical disability measured with the EDSS, the associations were weak at young ages but became stronger at 40–50 years of age and remained so at even older ages. Similar trends were observed for the MSIS-29 physical and psychological scales. One explanation for the weaker association with the EDSS at young ages might be that EDSS has a higher variability at low points and young ages.^28^ Another plausible explanation is that the plasticity and functional reorganization of the CNS, which effectively compensates for the lesion/atrophy development in young persons with MS, decreases with age.^29^ Thus, as the structural damage in the CNS increases and the compensatory mechanisms decreases with age, MS-related structural pathology will have an overt impact on clinical severity, and the disease will start to transition into the secondary progressive form. However, the lesion distribution may have different degrees of impact on EDSS scores and MRI metrics, respectively. In particular, this concerns spinal cord lesions, which may have a particular impact on the EDSS score.^30^ Nevertheless, our results likely reflect the conversion to secondary progressive MS, since the association remained stronger at old ages than at young ages for the EDSS and the T_2_-lesion volume analysis when removing persons with primary progressive MS and persons with relapses recorded within six months before the EDSS scoring was performed. Furthermore, the reported clinical conversion to secondary progressive MS occurred approximately at the same ages when the (absolute) effect sizes of the EDSS and MSIS-29 started to increase in the rolling regressions. Interestingly, the direction and strengths of the associations with SDMT differed greatly between MRI measures and across ages. This suggests that the age might be a more complex predictor of the associations between information processing speed and MRI measures in MS than the other clinical variables. The potential mechanisms of these age-dependent associations are of clinical interest and warrant further investigation. Furthermore, our findings are consistent with the recently proposed brain-age paradigm, where high brain-predicted age in persons with MS has been shown to associate with clinical progression.^10^

We found that low baseline cortical, subcortical, and thalamic grey matter fractions and, to a lesser degree, high baseline T_1_-lesion volumes at baseline were associated with future long-term accrual of physical disability and decreased information processing speed, although the effect sizes were moderate. These results confirm and extend the knowledge obtained from a previous multi-center study including 1214 persons with MS which showed that low baseline subcortical grey matter volumes were associated with shorter time to higher EDSS milestones.^6^ Previous studies including smaller cohorts (*N*_range_ = 35–234) have indicated a pivotal role of grey matter volumes for different domains of cognitive function in MS.^7,12,14,15^ Our findings of significant associations between low baseline cortical, subcortical, and thalamic grey matter volume fractions and deteriorating information processing speed corroborate those findings with, to our knowledge, one of the largest sample sizes to date. One previous study including 118 persons with secondary progressive MS did not find any cross-sectional or longitudinal MSIS-29 associations with lesion volumes or global/regional brain volumes.^31^ However, in the current substantially better powered study with a more diverse group of persons with MS, we could show that low baseline subcortical volume fractions associate with deteriorating MSIS-29 physical scores, while no significant association was observed for the lesion volumes. Taken together, our results highlight the importance of grey matter volumes in contrast to lesion volumes for different domains of disability progression in persons with MS. The functional importance of both cortical and subcortical grey matter atrophy for different measures of disability in MS has been highlighted by previous studies, reflecting that subcortical collections of nuclei, primarily the thalamus, are intimately connected with many cortical regions.^6,32^ Atrophy of cortical and subcortical grey matter regions in MS may be facilitated by anterograde and retrograde neurodegeneration of the connecting nerve tracts, although the exact underlying pathomechanisms are poorly understood. It has previously been shown that thalamic atrophy is associated with alterations in global thalamocortical functional connectivity, which may explain the observed associations between low volume fractions of the grey matter regions and poor clinical outcomes.^33^ The importance of grey matter atrophy for progression of MS is also underlined by an increasing amount of research suggesting that it could be a pivotal MRI marker of response to disease-modifying treatments (DMTs).^34^

Limitations of this study include that no healthy controls were included in this study, which could facilitate further interpretation of the SDMT associations. However, the lack of controls did not affect our ability to address the aim of the study to discriminate between the MRI metrics regarding the prediction of the clinical scores in persons with MS. Moreover, the SDMT only measures the information processing speed, and therefore, the associations with MRI volumetric measures might not be generalizable to other aspects of cognitive function. At the same time, it is a benefit that the test measures a clearly delimited cognitive domain, making it easier to interpret its correlations with neuroanatomical structures. We also did not correct for educational level in the SDMT analyses since this data was oftentimes lacking. However, it has been previously reported that educational level only accounts for a negligible amount of the variance of normalized SDMT scores, and validated normative scores which have been adjusted for education and calculated in sufficiently large cohorts in the Swedish population are lacking.^35^ Furthermore, since spinal cord MRI was not available, we were not able to directly assess the effect of spinal atrophy or lesion volumes on the clinical outcomes. However, removing persons with relapses within six months before the EDSS scoring did not substantially alter the results, indicating that spinal lesions did not unduly affect our results. The study participants were not screened for depressive disorders which potentially can impact cognitive performance. Nevertheless, the biological plausibility, temporality of predictors and outcome, and the consistency of our results with previous studies suggests that our results were not substantially confounded by depressive disorders. Other potential weaknesses were the missingness of clinical data in the study cohort, the use of different MRI scanners, and a technical variance in the FLAIR acquisition parameters. However, either these factors were adjusted for in the statistical models, sensitivity analyses did not indicate that these factors had a major impact on the results or, as for the 3D T_1_-weighted volumes, the same scanning protocol and voxel size were used for all scans. It should also be emphasized that while real-world data may pose challenges in terms of different types of bias, it also provides the opportunity to assess a wide range of clinical phenotypes.

### Conclusions

Our results suggest that the strength of the associations between MRI and clinical variables are age-dependent and, for physical disability and self-reported impact of MS, increase at the ages when most persons clinically convert to secondary progressive MS phenotype. This finding may eventually provide a new and more objective measure of the transition to progressive disease. Furthermore, baseline cortical and subcortical grey matter and thalamic fractions predict long-term worsening of information processing speed and physical disability, and these are stronger predictors than lesion volumes. Interestingly, baseline subcortical grey matter fractions also predict worsening of self-reported impact of MS, an outcome that has not been well studied in this context before. These findings confirm the role of grey matter volumes as potentially one of the most relevant imaging markers of disability progression in one of the largest real-world MS cohorts to date. Further studies are warranted to investigate the potential mechanisms of the age-dependent associations and to more conclusively determine whether atrophy can be used as a marker of treatment response.

## Supplemental Material

sj-docx-1-mso-10.1177_20552173221144230 - Supplemental material for Association between brain volume and disability over time in multiple sclerosis Click here for additional data file. Supplemental material, sj-docx-1-mso-10.1177_20552173221144230 for Association between brain volume and disability over time in multiple sclerosis by Thomas Moridi, Leszek Stawiarz, Kyla A McKay, Benjamin V Ineichen, Russell Ouellette, Daniel Ferreira, J-Sebastian Muehlboeck, Eric Westman, Ingrid Kockum, Tomas Olsson, Fredrik Piehl, Jan Hillert, Ali Manouchehrinia and Tobias Granberg in Multiple Sclerosis Journal – Experimental, Translational and Clinical

sj-docx-2-mso-10.1177_20552173221144230 - Supplemental material for Association between brain volume and disability over time in multiple sclerosis Click here for additional data file. Supplemental material, sj-docx-2-mso-10.1177_20552173221144230 for Association between brain volume and disability over time in multiple sclerosis by Thomas Moridi, Leszek Stawiarz, Kyla A McKay, Benjamin V Ineichen, Russell Ouellette, Daniel Ferreira, J-Sebastian Muehlboeck, Eric Westman, Ingrid Kockum, Tomas Olsson, Fredrik Piehl, Jan Hillert, Ali Manouchehrinia and Tobias Granberg in Multiple Sclerosis Journal – Experimental, Translational and Clinical

sj-docx-3-mso-10.1177_20552173221144230 - Supplemental material for Association between brain volume and disability over time in multiple sclerosis Click here for additional data file. Supplemental material, sj-docx-3-mso-10.1177_20552173221144230 for Association between brain volume and disability over time in multiple sclerosis by Thomas Moridi, Leszek Stawiarz, Kyla A McKay, Benjamin V Ineichen, Russell Ouellette, Daniel Ferreira, J-Sebastian Muehlboeck, Eric Westman, Ingrid Kockum, Tomas Olsson, Fredrik Piehl, Jan Hillert, Ali Manouchehrinia and Tobias Granberg in Multiple Sclerosis Journal – Experimental, Translational and Clinical
