# Supplementary material for: Association between brain volume and disability over time in multiple sclerosis
Source: Mult Scler J Exp Transl Clin. 2022 Dec 18;8(4):20552173221144230. doi: 10.1177/20552173221144230 (PMC9768834; doi:10.1177/20552173221144230)
Supplement: sj-docx-1-mso-10.1177_20552173221144230 - Supplemental material for Association between brain volume and disability over time in multiple sclerosis [file sj-docx-1-mso-10.1177_20552173221144230.docx]

**Supplementary material**

**Appendix I**

**1 Statistical analysis**

In order to assess how the MRI measures associated with the clinical variables across different ages of the study participants at baseline, rolling multiple regression models were used with the tibbletime package in R. The following candidate covariates were used: sex, age at MRI scan, age at clinical testing, age at onset of first symptoms, FLAIR sequence type (2D vs 3D) for the T_2_-lesion volumes, MS subtype (relapsing-onset defined as relapsing-remitting or secondary progressive MS vs. progressive-onset MS), total number of completed SDMTs (in the SDMT analysis) and treatment. Treatment exposure was defined as 1) the proportion of the time period between age at onset of MS and the first clinical score, during which the study participant had ongoing disease-modifying treatments (DMTs), and 2) ever versus never treatment with glucocorticoids during this time period. Exposure to platform DMTs (interferons, glatiramer acetate, teriflunomide and dimethyl fumarate), highly active DMTs (fingolimod, natalizumab, anti-CD20 therapies, alemtuzumab, daclizumab, cladribine, mitoxantrone and hematopoietic stem cell transplantation) and glucocorticoids were used as three separate covariates; continuous for DMTs and dichotomous for glucocorticoids. In a sensitivity analysis, persons with primary progressive MS and persons with a recorded relapse within six months before the clinical scores were excluded.

We assessed the associations between the baseline MRI and longitudinal clinical variables with linear mixed-effects models. Linear mixed-effects models allow for the longitudinal analysis of multiple data points from each individual while accounting for nested correlation structures within individuals and within MRI scanners. Importantly, these models can also account for an unequal number of data points between individuals or groups, and unequal time spacing between data points. In order to test whether the baseline brain volume fractions or lesion volumes predict change in clinical scores over time, we used an interaction term that included the baseline MRI predictor variable and the time after baseline for each clinical observation. Each baseline observation of the MRI variables was categorized as *high* or *low* depending on whether the value was above or below/equal to the median of the study population. Study subjects and MRI scanners were modeled as nested random effects with random slopes on time. EDSS raters were not included as a random effect since it resulted in less parsimonious models. Candidate fixed effect covariates included sex, time-varying age at MRI scan, time-varying age at clinical testing, age at onset of first symptoms, FLAIR sequence type (2D vs 3D) for the T_2_-lesion volumes, MS subtype (relapsing-onset vs. progressive-onset), total number of completed SDMTs (in the SDMT analysis) and treatment exposure. Treatment exposure was defined as 1) the proportion of the time period between each EDSS, SDMT and MSIS-29 score (and between age at onset of MS and the first score), respectively, during which the study participant had ongoing DMTs, and 2) ever versus never treatment with glucocorticoids during each time period. Exposure to platform DMTs, highly active DMTs and glucocorticoids as listed in the cross-sectional analysis were used as three separate covariates; continuous for DMTs and dichotomous for glucocorticoids. In order to assess the robustness of our results from the longitudinal analyses, we performed the following sensitivity analyses: 1) only including individuals with all four clinical variables available, 2) only including the MRI scanner for which the largest number of clinical data points were available andStepwise model reduction was performed to attain the most parsimonious models for each clinical variable in both the cross-sectional and longitudinal analyses.

**2 Data availability**

Data pertaining to the current study will be shared upon reasonable request. A data transfer agreement needs to be established between the institution requesting the data and Karolinska Institutet, in order to comply with the General Data Protection Regulation (GDPR) in Europe.
