# Supplementary material for: Association between brain volume and disability over time in multiple sclerosis
Source: Mult Scler J Exp Transl Clin. 2022 Dec 18;8(4):20552173221144230. doi: 10.1177/20552173221144230 (PMC9768834; doi:10.1177/20552173221144230)
Supplement: sj-docx-2-mso-10.1177_20552173221144230 - Supplemental material for Association between brain volume and disability over time in multiple sclerosis [file sj-docx-2-mso-10.1177_20552173221144230.docx]

**Appendix II**

**1 Predictors used in the final models for the main analyses**

**1.1 Rolling regression models of baseline MRI measures and clinical variables across ages**

Due to a larger sample size, a bandwidth of 100 persons was used for the EDSS rolling regressions, while a bandwidth of 30 persons was used for the other clinical variables. Stepwise reduction of multiple regression models including the entire age span of persons was used for covariate selection. MS subtype (relapsing-onset vs primary progressive MS) was not included as a covariate due to too few cases of primary progressive MS.

EDSS: N = 441. Age at onset was used as a covariate.

SDMT: N = 148. MRI scanner was used as a covariate.

MSIS-29 physical: N = 190. Age at onset, sex, MRI scanner and platform DMT exposure were used as covariates.

MSIS-29 psychological: N = 190. MRI scanner was used as a covariate.

**1.2 Associations between baseline MRI measures and longitudinal clinical variables**

In all models, study subjects and MRI scanners were used as nested random effects with random slopes on time of measurement of the clinical scores.

*EDSS*: N = 672. There were 5515 EDSS scores. Baseline age at MRI, age at onset, disease type (relapsing-onset vs primary progressive multiple sclerosis), highly active DMT exposure, MRI measure, time after baseline clinical examination and an MRI measure*time interaction term were used as fixed effects.

*SDMT*: N =779. There were 6011 SDMT scores. Baseline age at MRI, age at onset, total number of completed SDMTs, FLAIR sequence type (2D vs 3D), disease type (relapsing-onset vs primary progressive multiple sclerosis), highly active DMT exposure, MRI measure, time after baseline clinical examination and an MRI measure*time interaction term were used as fixed effects.

*MSIS-29 physical*: N = 820. There were 5497 MSIS-29 physical scores. Baseline age at MRI, baseline age at clinical examination, age at onset, sex, FLAIR sequence type (2D vs 3D), disease type (relapsing-onset vs primary progressive multiple sclerosis), platform and highly active DMT exposure, MRI measure, time after baseline clinical examination and an MRI measure*time interaction term were used as fixed effects.

*MSIS-29 psychological*: N = 820. There were 5497 MSIS-29 psychological scores. Baseline age at MRI, age at onset, sex, disease type (relapsing-onset vs primary progressive multiple sclerosis), platform and highly active DMT exposure, MRI measure, time after baseline clinical examination and an MRI measure*time interaction term were used as fixed effects.
