# Supplementary material for: Association between brain volume and disability over time in multiple sclerosis
Source: Mult Scler J Exp Transl Clin. 2022 Dec 18;8(4):20552173221144230. doi: 10.1177/20552173221144230 (PMC9768834; doi:10.1177/20552173221144230)
Supplement: sj-docx-3-mso-10.1177_20552173221144230 - Supplemental material for Association between brain volume and disability over time in multiple sclerosis [file sj-docx-3-mso-10.1177_20552173221144230.docx]

| Pulse sequence parameters |  | | | |
| --- | --- | --- | --- | --- |
| T1-weighted MPRAGE |  |  |  |  |
| Field strength, Tesla | 1.5 – 3.0 | | | |
| Voxel size, mm^3^ | 1.0×1.0×1.5 | | | |
| Repetition time, ms | 1350 – 1900 | | | |
| Echo time, ms | 3.02 – 7.00 | | | |
| Inversion time, ms | 300 – 1100 | | | |
| Flip angle, ^o^ | 9 – 15 | | | |
| T2-weighted FLAIR |  |  |  |  |
| Field strength, Tesla | 1.5 – 3.0 | | | |
| Voxel size, mm^3^ | (0.5 – 1.0)×(0.5 – 1.0)×(1.0 – 3.0) | | | |
| Repetition time, ms | 5000 – 6000 | | | |
| Echo time, ms | 284 – 388 | | | |
| Inversion time, ms | 1800 – 2100 | | | |
| Flip angle, ^o^ | 120 (variable) | | | |

**Supplementary Table 1. Magnetic resonance imaging acquisition and post-processing parameters.**

Abbreviations: FLAIR, fluid-attenuated inversion recovery; MPRAGE, magnetization-prepared rapid gradient echo; ms, milliseconds.

**
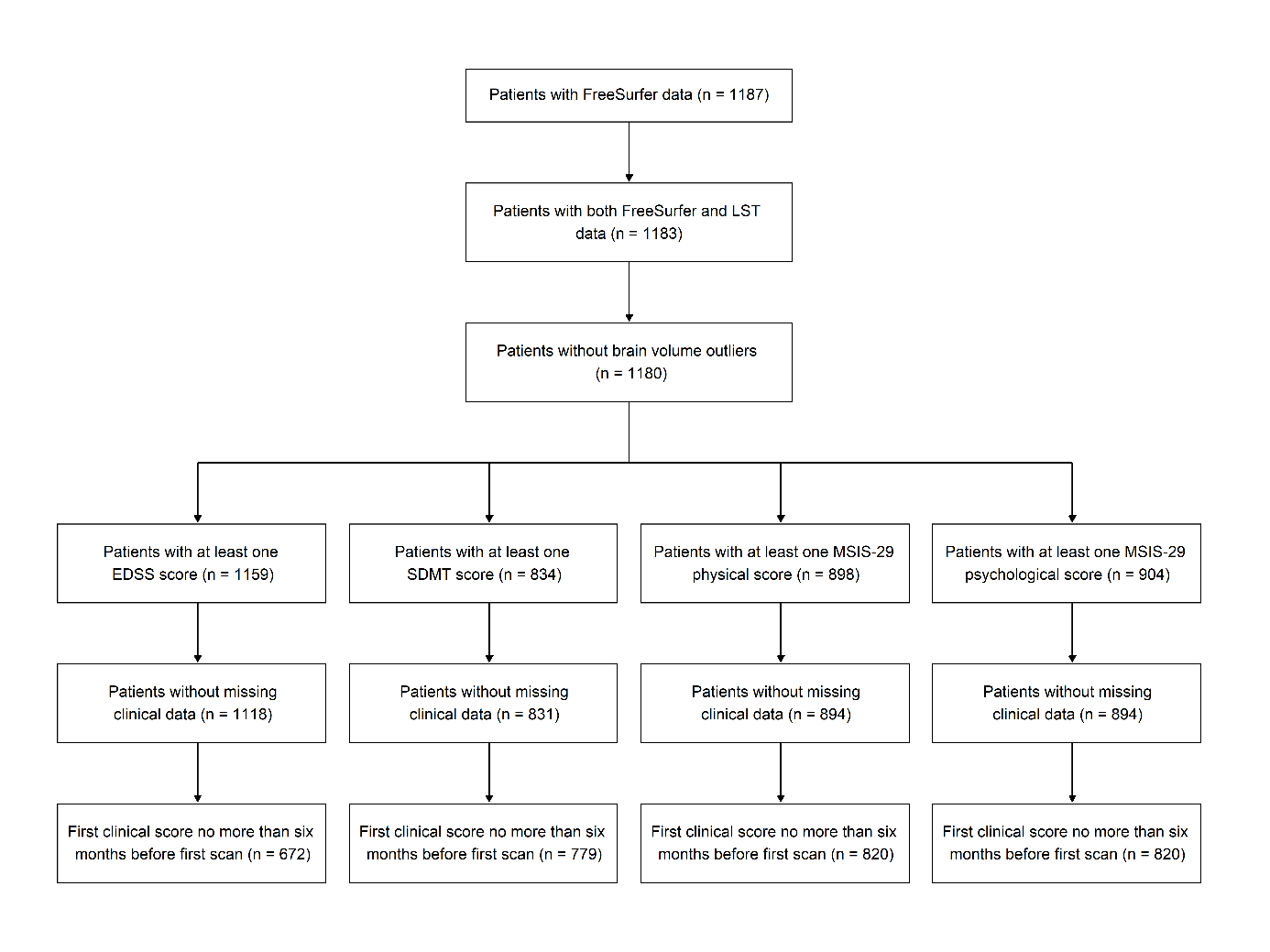
**

**Supplementary Figure 1. Flowchart of study participant inclusion/exclusions and missing data**

Abbreviations: EDSS, Expanded Disability Status Scale; LST, Lesion Segmentation Tool; MSIS-29, Multiple Sclerosis Impact Scale 29; SDMT, Symbol Digit Modalities Test.

**
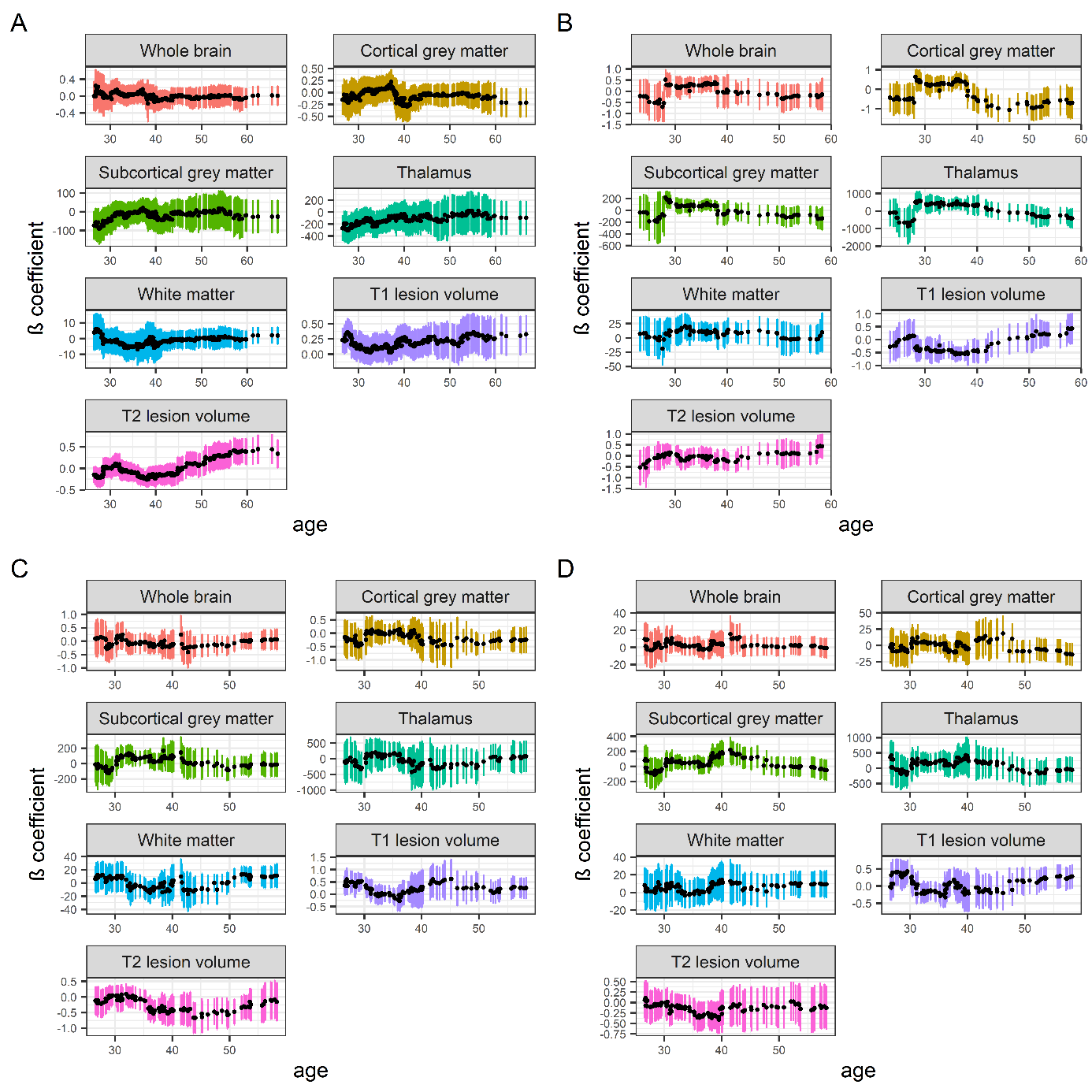
**

**Supplementary Figure 2. Sensitivity analysis of rolling regression models**

Rolling multiple regression models of how baseline MRI brain volume fractions and lesion volumes associate with clinical variables across different ages of the persons with MS. Persons with primary progressive MS and persons with a recorded relapse within six months before the clinical scores were excluded. Bandwidths of 70, 20 and 30 persons were used for the EDSS, SDMT and MSIS-29 rolling regressions, respectively. The colored vertical bars represent the 95 % confidence intervals for each regression. Abbreviations: DMT, Disease-modifying treatment; EDSS, Expanded Disability Status Scale; MRI, Magnetic Resonance Imaging; MS, multiple sclerosis; MSIS-29, MS Impact Scale 29; SDMT, Symbol Digit Modalities Test.

A) EDSS. N = 327. Age at onset was used as a covariate.

B) SDMT. N = 103. MRI scanner was used as a covariate.

C) MSIS-29 physical. N = 143. Age at onset, sex, MRI scanner and highly active DMT exposure were used as covariates.

D) MSIS-29 psychological. N = 143. MRI scanner was used as a covariate.

**
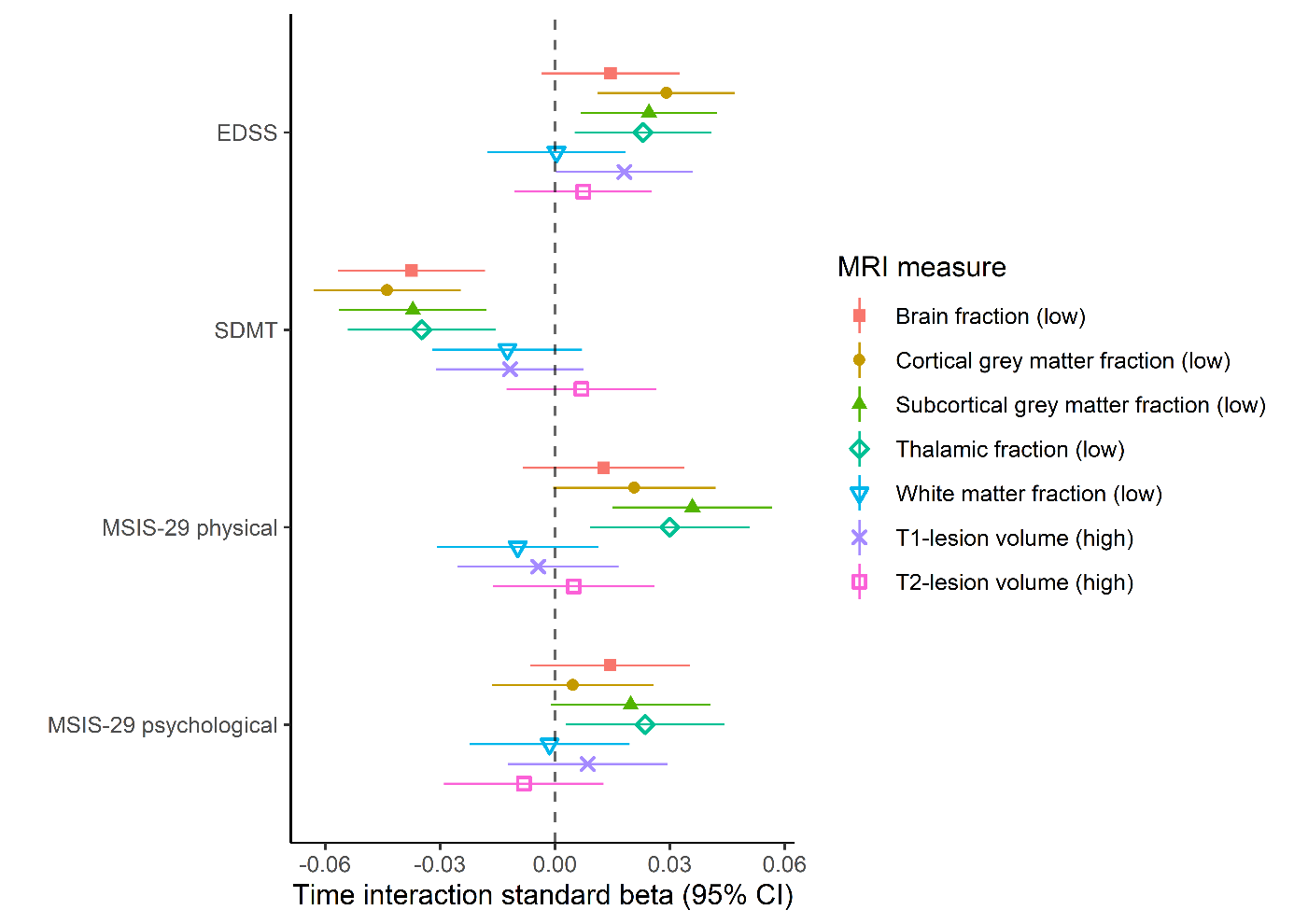
**

**Supplementary Figure 3. Forest plot of linear mixed-effects model complete set analysis of longitudinal clinical measures and baseline MRI measures with time interaction**

The analysis only included individuals with all four clinical variables available (N = 486). In all models, study subjects and MRI scanners were used as nested random effects with random slopes on time of measurement of the clinical scores. Abbreviations: CI, confidence interval; DMT, Disease-modifying treatment; EDSS, Expanded Disability Status Scale; FLAIR, fluid-attenuated inversion recovery; MRI, Magnetic Resonance Imaging; MSIS-29, Multiple Sclerosis Impact Scale 29; SDMT, Symbol Digit Modalities Test

*EDSS*: There were 4543 EDSS scores. Baseline age at MRI, age at onset, disease type (relapsing-onset vs primary progressive multiple sclerosis), highly active DMT exposure, MRI measure, time after baseline clinical examination and an MRI measure*time interaction term were used as fixed effects.

*SDMT*: There were 3873 SDMT scores. Baseline age at MRI, age at onset, total number of completed SDMTs, FLAIR sequence type (2D vs 3D), disease type (relapsing-onset vs primary progressive multiple sclerosis), highly active DMT exposure, MRI measure, time after baseline clinical examination and an MRI measure*time interaction term were used as fixed effects.

*MSIS-29 physical*: There were 3549 MSIS-29 physical scores. Baseline age at MRI, baseline age at clinical examination, age at onset, sex, FLAIR sequence type (2D vs 3D), disease type (relapsing-onset vs primary progressive multiple sclerosis), platform and highly active DMT exposure, MRI measure, time after baseline clinical examination and an MRI measure*time interaction term were used as fixed effects.

*MSIS-29 psychological*: There were 3549 MSIS-29 psychological scores. Baseline age at MRI, age at onset, sex, disease type (relapsing-onset vs primary progressive multiple sclerosis), platform and highly active DMT exposure, MRI measure, time after baseline clinical examination and an MRI measure*time interaction term were used as fixed effects.

**
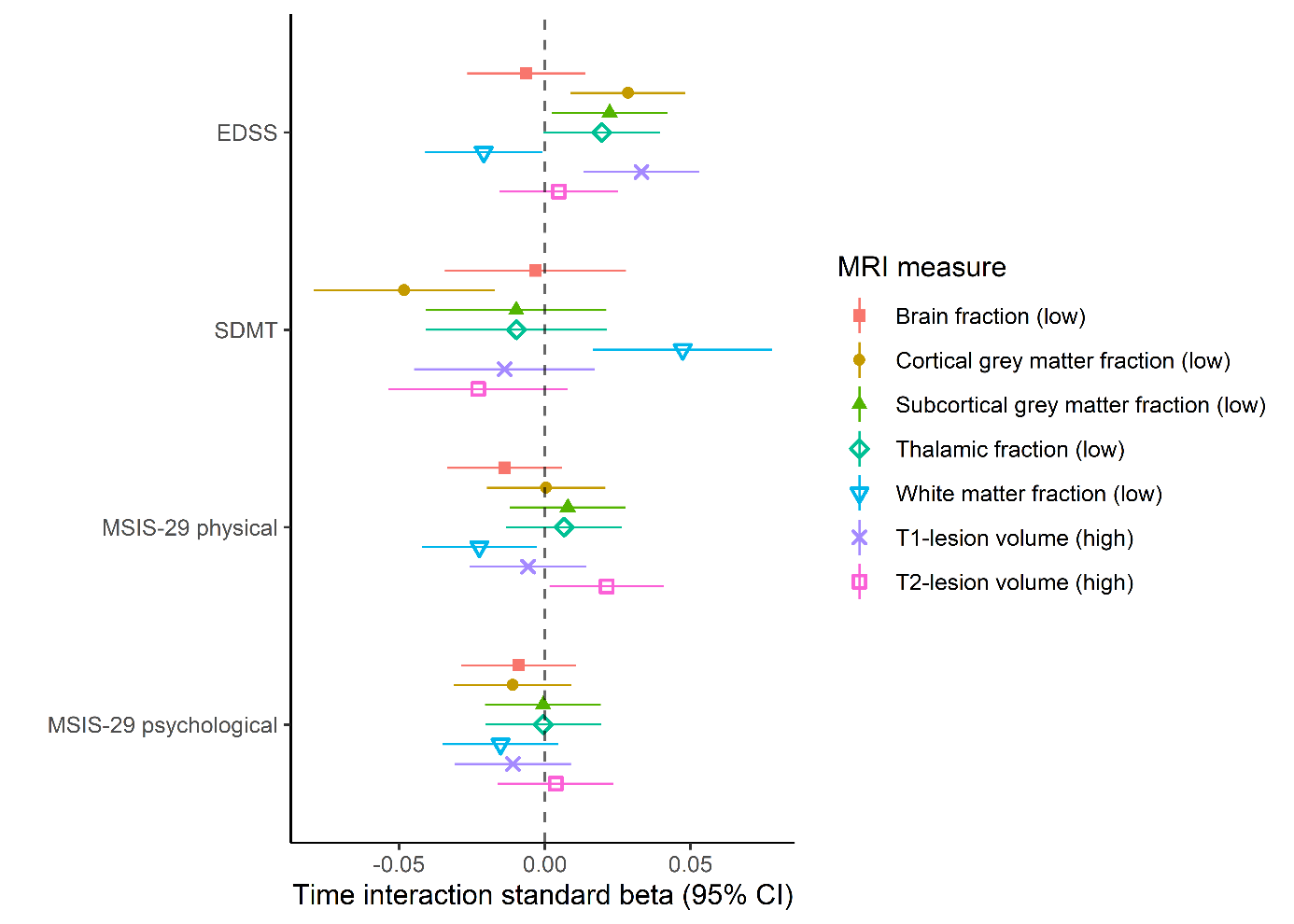
**

**Supplementary Figure 4. Forest plot of linear mixed-effects model analysis of longitudinal clinical measures and baseline Vision MRI measures with time interaction**

The analysis only included individuals with all four clinical variables available (N = 486). In all models, study subjects and MRI scanners were used as nested random effects with random slopes on time of measurement of the clinical scores. Abbreviations: CI, confidence interval; DMT, Disease-modifying treatment; EDSS, Expanded Disability Status Scale; FLAIR, fluid-attenuated inversion recovery; MRI, Magnetic Resonance Imaging; MSIS-29, Multiple Sclerosis Impact Scale 29; SDMT, Symbol Digit Modalities Test

*EDSS*: N = 234. There were 2656 EDSS scores. Baseline age at MRI, age at onset, disease type (relapsing-onset vs primary progressive multiple sclerosis), highly active DMT exposure, MRI measure, time after baseline clinical examination and an MRI measure*time interaction term were used as fixed effects.

*SDMT*: N = 251. There were 2068 SDMT scores. Baseline age at MRI, age at onset, total number of completed SDMTs, disease type (relapsing-onset vs primary progressive multiple sclerosis), highly active DMT exposure, MRI measure, time after baseline clinical examination and an MRI measure*time interaction term were used as fixed effects.

*MSIS-29 physical*: N = 309. There were 2010 MSIS-29 physical scores. Baseline age at MRI, baseline age at clinical examination, age at onset, sex, disease type (relapsing-onset vs primary progressive multiple sclerosis), platform and highly active DMT exposure, MRI measure, time after baseline clinical examination and an MRI measure*time interaction term were used as fixed effects.

*MSIS-29 psychological*: N = 309. There were 2010 MSIS-29 psychological scores. Baseline age at MRI, age at onset, sex, disease type (relapsing-onset vs primary progressive multiple sclerosis), platform and highly active DMT exposure, MRI measure, time after baseline clinical examination and an MRI measure*time interaction term were used as fixed effects.
